# Mass spectrometry analysis of human tear fluid biomarkers specific for ocular and systemic diseases in the context of 3P medicine

**DOI:** 10.1007/s13167-021-00265-y

**Published:** 2021-12-03

**Authors:** Xianquan Zhan, Jiajia Li, Yuna Guo, Olga Golubnitschaja

**Affiliations:** 1 Shandong Key Laboratory of Radiation Oncology, Shandong Cancer Hospital and Institute, Shandong First Medical University, 440 Jiyan Road, Jinan, 250117 Shandong China; 2 Medical Science and Technology Innovation Center, Shandong First Medical University, 6699 Qingdao Road, Jinan, 250117 Shandong China; 3 Gastroenterology Research Institute and Clinical Center, Shandong First Medical University, 38 Wuying Shan Road, Jinan, Shandong 250031 People’s Republic of China; 4 Key Laboratory of Cancer Proteomics of Chinese Ministry of Health, Xiangya Hospital, Central South University, 87 Xiangya Road, Changsha, 410008 Hunan China; 5 Predictive, Preventive and Personalised (3P) Medicine, Department of Radiation Oncology, University Hospital Bonn, Rheinische Friedrich-Wilhelms-University of Bonn, Sigmund-Freud-Str 25, 53105 Bonn, Germany

**Keywords:** Tear fluid, Mass spectrometry (MS), Biomarker panel, Patterns, Predictive preventive personalized medicine (3PM/PPPM), Novel targets, Differential proteomics, MALDI-TOF, Electrophoretic techniques, 2D-PAGE, SDS-PAGE, In-gel digestion, HPLC, Microarrays, ELISA, Sub-optimal health, Ocular pathologies, Systemic disorders, Ocular allergy, Dry eye, Sicca syndrome, Diabetic retinopathy, Glaucoma, Meibomian gland dysfunction, Thyroid-associated ophthalmopathy, Breast cancer, Prostate cancer, Melanoma, Retinoblastoma, Multiple sclerosis, Parkinson’s disease, Sjögren syndrome, Sample processing, Schirmer test, MMP-9, Antimicrobial compounds, Autoantibody, Inflammatory cytokines, S100, Calgranulin, Post-translational modification (PTM), Individualized patient profiling, Personalized services, Socio-economic impacts, Healthcare economy, Cost-efficacy, Pandemic, COVID-19

## Abstract

Over the last two decades, a large number of non-communicable/chronic disorders reached an epidemic level on a global scale such as diabetes mellitus type 2, cardio-vascular disease, several types of malignancies, neurological and eye pathologies—all exerted system’s enormous socio-economic burden to primary, secondary, and tertiary healthcare. The paradigm change from reactive to predictive, preventive, and personalized medicine (3PM/PPPM) has been declared as an essential transformation of the overall healthcare approach to benefit the patient and society at large. To this end, specific biomarker panels are instrumental for a cost-effective predictive approach of individualized prevention and treatments tailored to the person. The source of biomarkers is crucial for specificity and reliability of diagnostic tests and treatment targets. Furthermore, any diagnostic approach preferentially should be noninvasive to increase availability of the biomaterial, and to decrease risks of potential complications as well as concomitant costs. These requirements are clearly fulfilled by tear fluid, which represents a precious source of biomarker panels. The well-justified principle of a “sick eye in a sick body” makes comprehensive tear fluid biomarker profiling highly relevant not only for diagnostics of eye pathologies but also for prediction, prognosis, and treatment monitoring of systemic diseases. One prominent example is the Sicca syndrome linked to a cascade of severe complications that include dry eye, neurologic, and oncologic diseases. In this review, protein profiles in tear fluid are highlighted and corresponding biomarkers are exemplified for several relevant pathologies, including dry eye disease, diabetic retinopathy, cancers, and neurological disorders. Corresponding analytical approaches such as sample pre-processing, differential proteomics, electrophoretic techniques, high-performance liquid chromatography (HPLC), enzyme-linked immuno-sorbent assay (ELISA), microarrays, and mass spectrometry (MS) methodology are detailed. Consequently, we proposed the overall strategies based on the tear fluid biomarkers application for 3P medicine practice. In the context of 3P medicine, tear fluid analytical pathways are considered to predict disease development, to target preventive measures, and to create treatment algorithms tailored to individual patient profiles.

## Introduction

Tears, also known as tear fluid or tear film, are the outermost thin liquid layer that covers the ocular surface epithelial cells to form an anterior meniscus component at the eyelid margins [1, 2]. Tears serve as a crucial optical smooth surface to refract light from air onto the retina, and are an important and complex body fluid [1]. The intricate secretion and structure of tear film derive from its various secretory units, including lacrimal glands, meibomian glands, accessory lacrimal glands, sebaceous glands of Zeis and Moll, and corneal and conjunctival cells [3]. In addition, an ultrafiltrate of blood contributes to the composition of tear fluid [4]. Under normal conditions, reflex tears as well as tear flow derive from the main lacrimal glands, whereas basal tears might be triggered from accessary lacrimal glands [5, 6]. Generally, reflex tear secretion is triggered by activation of the corneal nerves due to an irritating stimulus [7]. Normally, the amount of tear fluid is 5–10 ml [8] with a secretion rate of ~ 1.2 μl per minute [5, 9] and a turnover rate of ~ 16% per minute [5].

Tear film serves as the first barrier between the external environment and the eye to play a crucial role to protect and maintain the health of the ocular surface [10]. The primary functions of tear film include the following: (i) lubricate and lessen the friction between the eye surface and the eyelids during blinking, and to moisten the ocular surface between the palpebral and bulbar conjunctiva [11, 12], (ii) provide oxygen and electrolytes to the cornea that are essential for corneal metabolism [2, 13], (iii) increase the refractive power of the eye because a healthy tear film might enhance the optical quality [14], and (iv) protect the eye from environment debris and bacterial infection with a physical flush and antibacterial constituents such as lysozyme and secretory immunoglobin A (sIgA) [15–17].

**Fig. 1:**
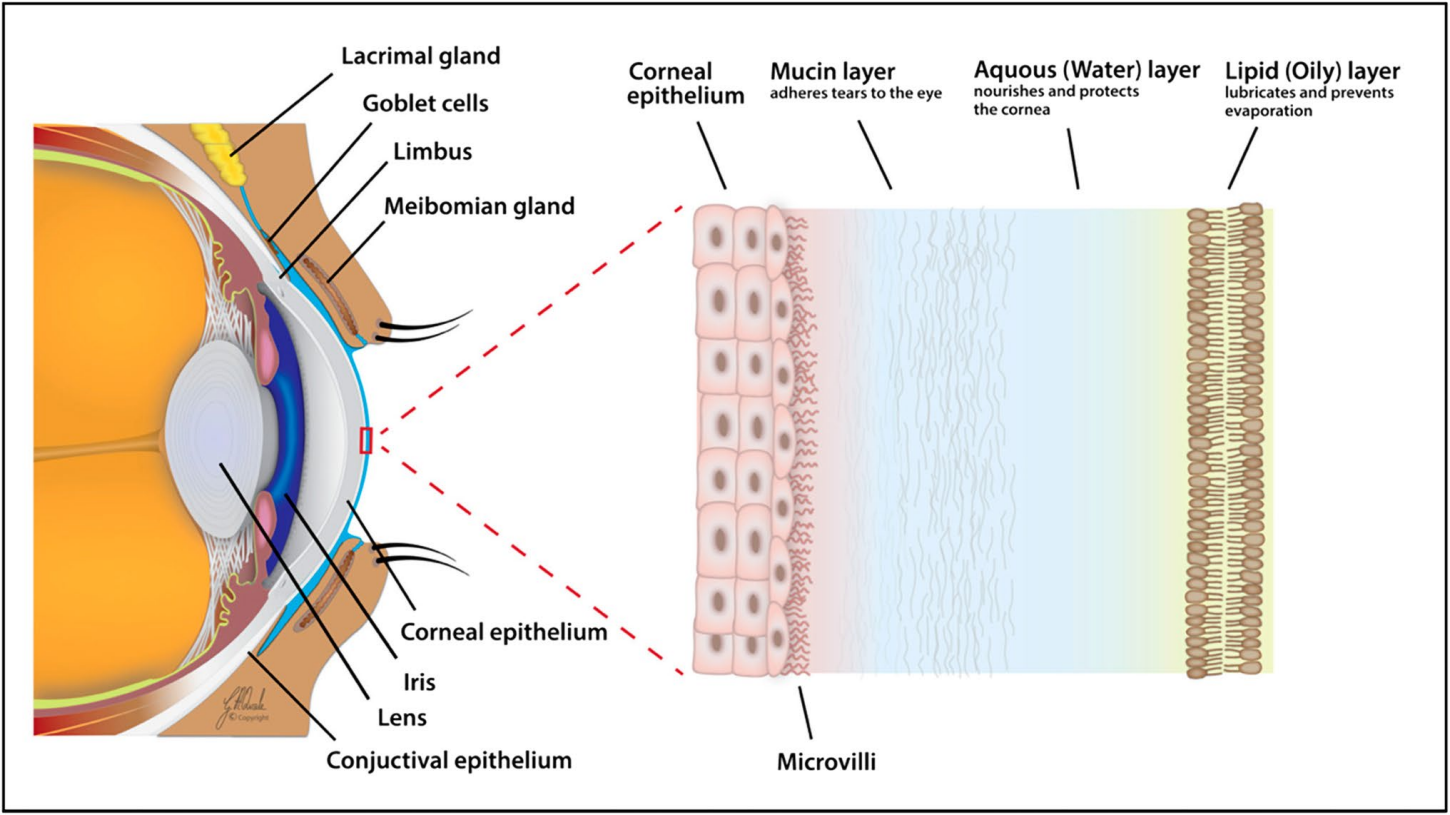
The three-layered structure of the tear film: an outer lipid layer at the air surface, an intermediate aqueous layer, and an inner mucus layer on the epithelial. Reproduced from Yazdani et al. [2], with copyright permission from MDPI publisher open access article, copyright 2019

There is no single conclusive representation for the exact structure of tear fluid. The classical model of tear film consists of three layers [1, 18]: a superficial lipid layer, a middle aqueous layer that contains primarily proteins and metabolites, and an inner mucous/glycocalyx layer that contacts the ocular surface epithelial cells (Fig. 1). Fluorescent quantum dots, which are specially modified to be either lipophilic or hydrophilic, have been applied to study the dynamics of tear film [19]. The eye surface and eyelid margin are distinct from each other because of the dispersion patterns of the lipophilic and hydrophilic quantum dots; those dots suggest and support the three-layer model hypothesis of distinct lipid, aqueous, and mucin layers in the tear film [19, 20]. Furthermore, a recent new precorneal tear film model proposed that lipid layer could be further divided into two sublayers—the inner polar lipid layer with intercalated proteins and the outer non-polar lipid layer [21]. The components of three layers have their own major sources: (i) The lipid layer components are mainly generated from the meibomian glands, which contribute to maintain stability of the tear film and prevent fast evaporation of the underlying aqueous layer [22]; (ii) the aqueous layer components are mainly secreted by main and accessory lacrimal glands. In a general way, the compositions of the aqueous layer are mainly electrolytes (sodium, potassium, calcium, phosphate, bicarbonate, etc.), proteins/peptides (enzymes, neuropeptides, growth factors, etc.), and small-molecule metabolites (amino acids, urea, glucose, etc.) [23]; (iii) the primary source of the mucin layer is goblet cells in the conjunctiva; and conjunctival epithelial cells, corneal cells, and even lacrimal glands might also play a part to generate mucin layer [1]. Mucins, a typically large glycoprotein, contain O-linked carbohydrates and a protein core [1]. The total tear film thickness was initially considered to be between 7 and 9 mm [11], and to include a 0.1-μm lipid layer, 7-μm aqueous layer, and 0.05-μm mucous layer [24]. A current study estimated the tear film thickness range between 3 and 11 mm [25].

Despite relatively small amount, tear fluid is quite a complex biological liquid mixture that contains proteins/peptides, electrolytes, lipids, and small-molecule metabolites as mentioned above [26]. The composition of tears could reflect the physiological condition of some underlying organs and systems [1]. Therefore, it is not hard to understand why the tear fluid is extensively helpful to evaluate health and disease states, and it is a significant source of biomarkers for not only objective analysis of ocular diseases such as dry eye [2, 11], keratoconus [12, 27], and blepharitis [28, 29], but also of systemic diseases such as cancer [30, 31] and multiple sclerosis (MuSc) [32].

This review discusses the main compositions of the tear film, recapitulates the proteomic strategies that have been applied to characterize tear proteins/peptides, and presents a brief summary of some biomarkers from discovery to clinical application for some diseases. Mass spectrometry plays a critical role in those studies.

## Composition of the tear film

### Protein composition of the tear film

Protein concentrations in tear fluid range from 6 to 11 mg/ml [33], which might be due to the different tear-collection approaches (Schirmer’s strip [34, 35], eye flush [36], glass capillary tube [37]), various protein assay methods (Bradford [38], Lowry method [39]), or types of collected tears (reflex, or non-stimulated tears) [5]. The tear film contains a complex mixture of proteins such as enzymes, neuropeptides, and protective proteins. In addition, human tear protein variety and protein levels might differ with respect to the sexes [40, 41], age [41, 42], and diurnal variation (daytime changes) [43, 44]. The major tear proteins can be divided into several categories: (i) proteins produced by the lacrimal glands, including lysozyme, lactoferrin, lipocalin, epidermal growth factor, and IgA; and (ii) a small proportion of serum proteins derived from the conjunctival capillaries, including albumin, transferrin, immunoglobulin G (IgG), and immunoglobulin M (IgM) [45]. Primary tear proteins are lactoferrin and lysozyme [46] that are secreted by the lacrimal acinar cells; lysozyme is the most-abundant tear protein [46] with concentrations of ~ 1.3 mg/ml in tear fluid [47]. These two proteins are known to perform antibacterial functions in the body [48]. Tear lipocalin is another significant protein in tear fluid that is combined with cholesterol, fatty alcohols, fatty acids, glycolipids, and the least-soluble lipid aqueous solution, which significantly enhances the solubility of these lipids in aqueous solution [49]. In addition, the rapid developments of proteomics and mass spectrometry significantly impact the elucidation of protein structures, activities, functions, and interactions in tear fluid [50]:

#### The total protein content

**Fig. 2:**
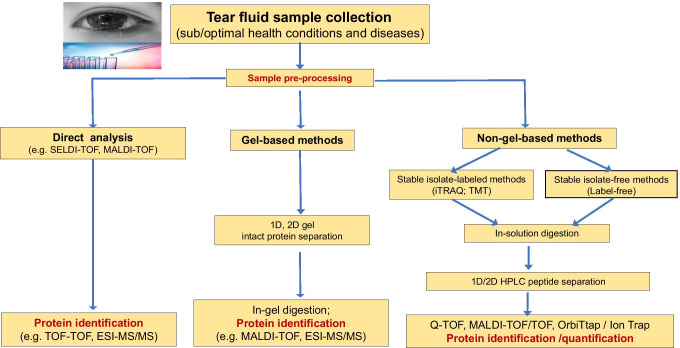
The main workflow for current mass spectrometry-based tear proteomics techniques. 1D: one dimensional. 2D: two dimensional. SELD: surface-enhanced laser desorption/ionization. TOF: time of flight. MALDI: matrix-assisted laser desorption ionization. ESI: electrospray ionization. MS/MS: tandem mass spectrometry. Q-TOF: quadrupole time of flight. iTRAQ: isobaric tags relative or absolute quantification. TMT: tandem mass tags

**Fig. 3:**
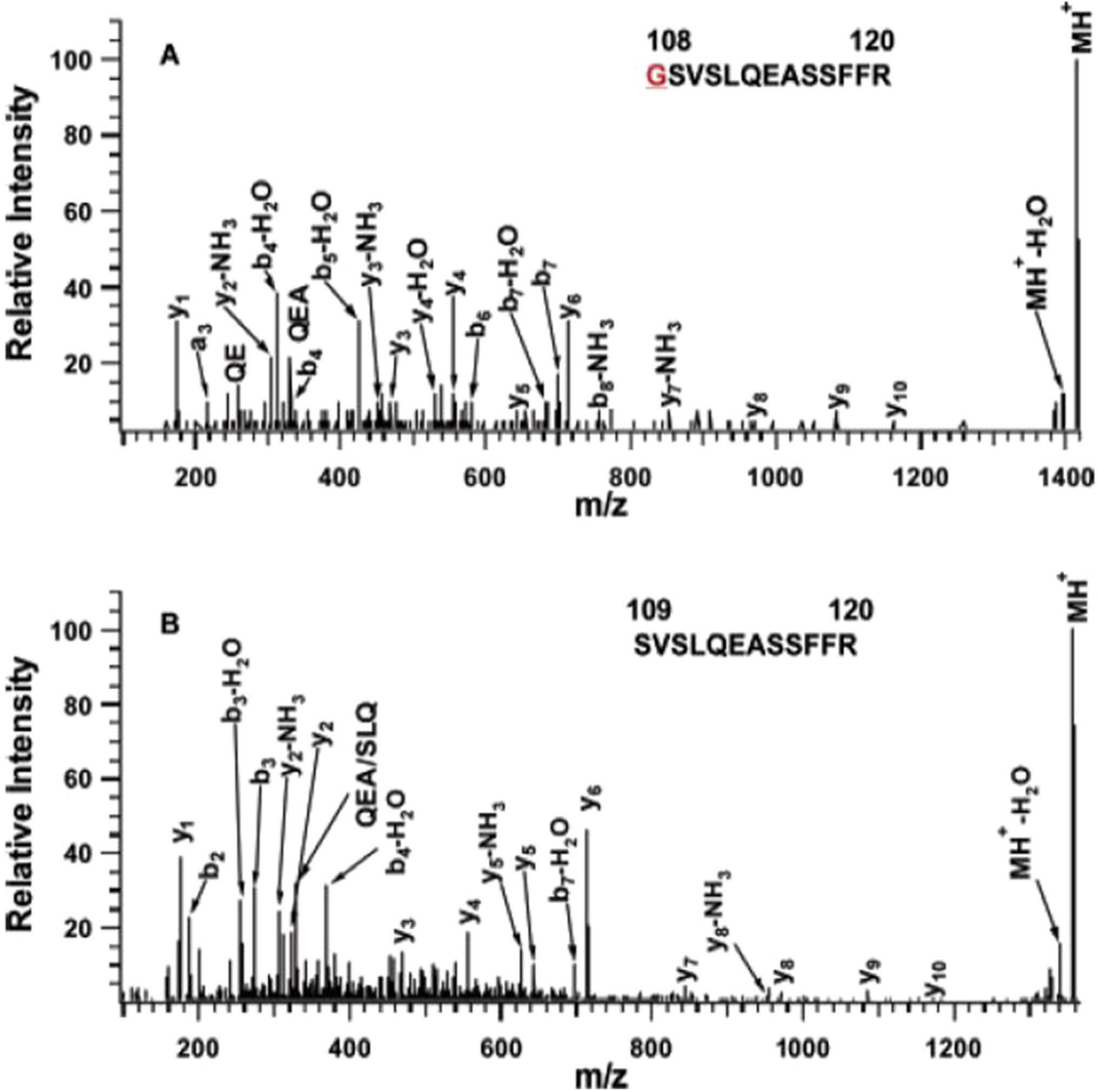
MALDI MS/MS analysis of two tryptic peptides derived from proline-rich protein 4 (Q16378) in human tears. Reproduced from Li N et al. [53], with copyright permission from American Chemistry Society publisher, copyright 2005

Multiple proteomics strategies have identified a large number of tear proteins in the last decade to expand the understanding of tear proteins (Fig. 2) [20, 51]. One of the earliest studies to identify the protein composition and content of normal human reflex tears has identified 6 different proteins (lipocalin, lactotransferrin, lysozyme, zinc-alpha-2 glycoprotein, cystatin S, and cystatin SN) with two-dimensional gel electrophoresis (2DGE) [52]. Another study focused on a gel-based and in-solution-based research strategy, used various ionization and acquisition approaches to identify and characterize tear protein content, and identified 54 proteins with ~ 5 μl sample volume of tears that were collected under normal circumstances in a clinical laboratory [53]. A total of 60 proteins were identified from the tears of pterygium patients via reverse phase high-performance liquid chromatography-tandem mass spectrometry (RP–HPLC–MS/MS) [54]. Furthermore, a substantial contribution to the tear protein database was made by De Souza and colleagues, who identified 491 different proteins via gel-based and gel-free methods and two types of MS methods [55]. Also, studies found 80–90% of the tear protein content is characterized by a small group of proteins that contain lipocalin, lysozyme, lactoferrin, sIgA, and serum albumin [55, 56]. A few years later, more studies extensively expanded the tear proteome database. For example, Zhou et al. identified 1543 proteins with strong cation exchange (SCX) chromatography-LC–MS/MS [51], and a later optimized proteomic analysis identified 1526 tear proteins with Schirmer's strips with 2D LC–MS/MS [57]. The representative MS/MS analyses of two tryptic peptides GSVSLQEASSFFR (position 108–120) and SVSLQEASSFFR (position 109–120) derived from proline-rich protein 4 (Q16378) in human tear fluid are shown (Fig. 3). For the tryptic peptide GSVSLQEASSFFR (position 108–120), the excellent b-ion and y-ion series (y_1_, y_2_-NH_3_, y_3_-NH_3_, y_3_, y_4_, y_4_-H_2_O, y_5_, y_6_, y_7_-NH_3_, y_8_, y_9_, y_10_, b_4_, b_4_-H_2_O, b_5_-H_2_O, b_6_, b_7_, b_7_-H_2_O, and b_8_-NH_3_) are obtained, with excellent signal-to-noise (S/N) ratio. For another peptide SVSLQEASSFFR (position 109–120), the excellent b-ion and y-ion series (y_1_, y_2_-NH_3_, y_2_, y_3_, y_4_, y_5_-NH_3_, y_5_, y_6_, y_7_, y_8_, y_8_-NH_3_, y_9_, y_10_, b_2_, b_3_-H_2_O, b_4_-H_2_O, and b_7_-H_2_O) are obtained, with excellent signal-to-noise (S/N) ratio. The two sequenced peptides are assigned to proline-rich protein 4 (Q16378) in human tears. The similar method was used to identify other tear proteins, and a brief summary of the main representative studies and their achievements about tear proteomics are displayed (Table 1).

**Table 1:** Proteomic procedures currently used for the human tear fluid analysis

Publication	Sample pre-processing (gel or HPLC)	MS type used	Number of identified proteins	Disease/health condition
[52]	2D-PAGE	None (Edman sequencing)	6	Healthy individuals
[53]	SDS-PAGE + in-gel digestion + online RP	LCQ Deca (Thermo), reflex III	54	Healthy individuals
		MALDI-TOF (Bruker)		
[54]	RP-HPLC + in-solution digestion	QSTAR XL (Applied Biosystems)	60	Pterygium surgery
[55]	1D-SDS-PAGE + in-gel digestion + online RP	LTQ-FT, LTQ-Orbi (Thermo)	491	Closed-eye tears
[21, 58]	SDS-PAGE + in-gel digestion	Finnigan LTQ-MS (Thermo)	97	Healthy individuals
[59]	iTRAQ + online 2D SCX-RP	2D-Nano-LC-nano-ESI–MS/MS(DIONEX)	93	Dry eye
[55]	Hydrazide enrichment	2D-Nano-LC-nano-ESI–MS/MS(DIONEX)	43 N-linked glycoproteins	Climatic droplet keratopathy
	Online 2D SCX-RP			
[60]	iTRAQ + online 2D SCX-RP	2D-Nano-LC-nano-ESI–MS/MS(DIONEX)	124	Glaucoma medication
[1]	2D-in-solution digestion + offline SCX	Triple TOF 5600 (ABsciex)	1543	Healthy individuals
[61, 62]	1D-SDS-PAGE + in-gel digestion + RP-RP capillary HPLC	MALDI TOF/TOF (Bruker)	267	Dry eye
				Contact lens wearers
[57]	In-solution digestion + offline SCX	LTQ Orbitrap XL (Thermo)	1526	Healthy individuals
[63]	Label-free + APEX quantitative proteomics tool	SYNAPT HDMS-MS (Waters)	603	Ocular surface diseases
[64]	RP-HPLC + in-solution digestion	nanoLC/Q-TOF–MS/MS	1031	Dry eye

#### The natural peptide content

Peptides in human tear fluid are mainly derived from natural processes that their corresponding protein precursors are degraded by specific enzymes. These peptides would be significantly characterized for the corresponding human tear proteins, and also these peptides might have bioactivities and specific functions, including antimicrobial activities or intercellular signaling [65–67]. However, information on these tear fluid peptides is still scarce relative to the current protein database [68]. One study identified 30 endogenous tear peptides from proline-rich protein 4 and polymeric immunoglobulin receptor in human reflex tears with LC-matrix-assisted laser/desorption ionization-time of flight-mass spectrometry (LC-MALDI-TOF-TOF–MS) [69]. Another study identified 234 peptides in a human basal tear sample, which were derived from 25 proteins, with MS analysis in different ion-fragment models including collision-induced dissociation (CID), electron transfer dissociation (ETD), and higher energy collision dissociation (HCD) [70]. For this study, one of the significant achievements was that two identified endogenous peptides from the extracellular glycoprotein lacritin had antimicrobial activities [70]. Those 234 endogenous peptides formed the current largest peptide dataset that are naturally existed in human basal tears.

#### Post-translational modifications of the tear proteins

**Fig. 4:**
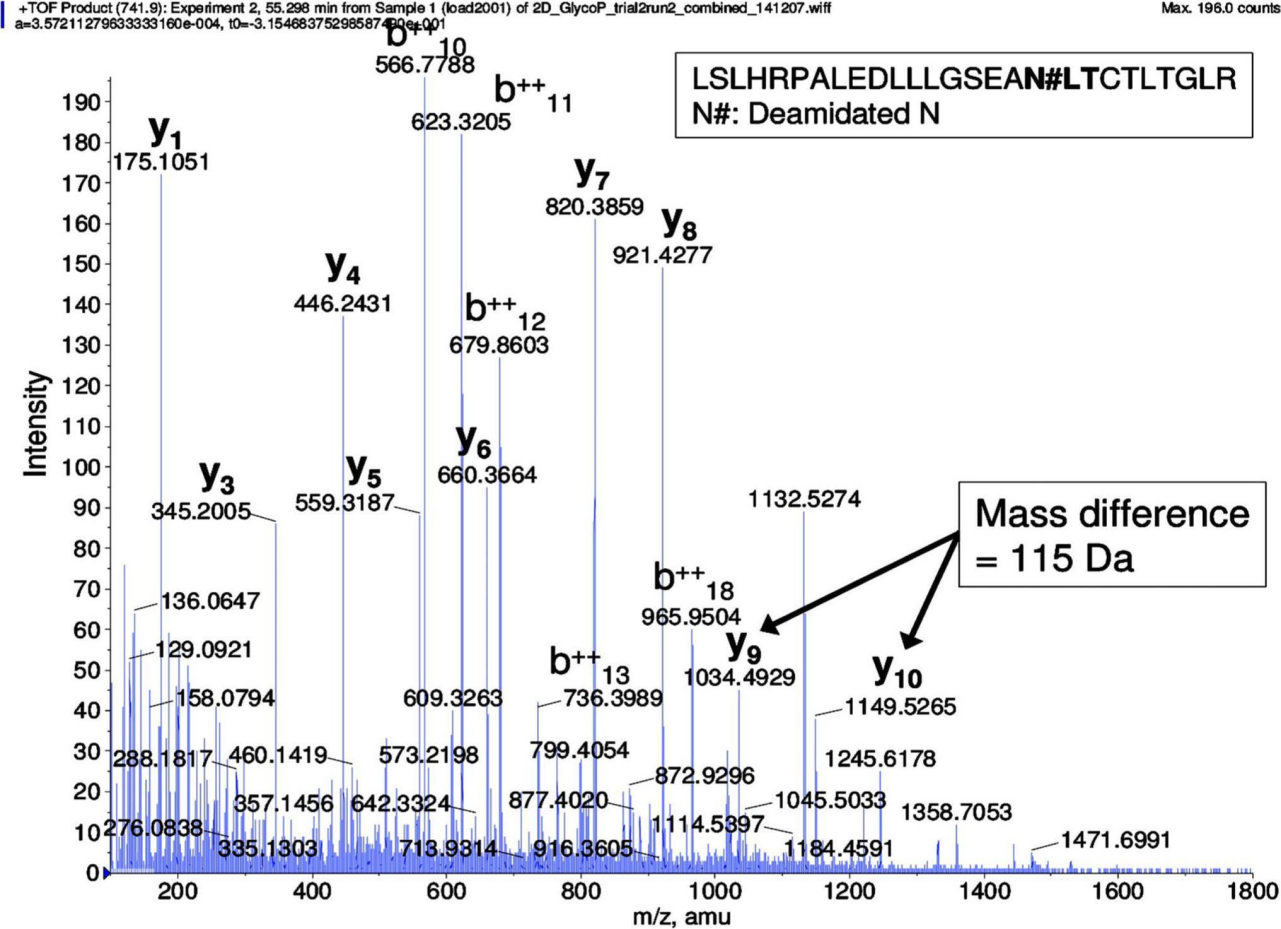
MS/MS spectrum of N-linked glycopeptide fragment LSLHRPALEDLLLGSEAN#LTCTLTGLR ([M + 4H]^4+^, *m*/*z* = 741.9) derived from SNC66 protein in tear fluid of climatic droplet keratopathy. N# represents the N-glycosylation site and the consensus motif of N-glycosylation is in bold. The mass difference of 115 Da between y9 and y10 ion confirms the deamidation of asparagine in the peptide. Reproduced from Zhou et al. [77], with copyright permission from American Chemistry Society publisher, copyright 2009

Protein post-translational modifications (PTMs) are defined as a modification of one or more amino acids of peptides by addition of a specific group (such as phosphate group) to lead to changes of protein functions and to allow the cell to regulate protein activities [71]. Consequently, PTMs play an important role in the regulation of the external conditions or internal states. Individually and cooperatively, PTMs can affect numerous properties of proteins, such as the subcellular location, protein interactions, and enzymatic activities [71]. In spite of the great interest in the PTMs on the aspect of protein functions and tens of thousands of modification sites identified and localized in proteins [72], little information has been obtained to characterize many PTMs in tear proteins. One study identified two variants of malignant brain tumor 1 protein (DMBT1) isoforms, which are the major high-molecular-mass glycoproteins in human tears, with low-percentage sodium dodecyl sulfate–polyacrylamide gel electrophoresis (SDS-PAGE) [73]. Advances in tear protein PTMs have been obtained from a study [53] that demonstrated the phosphorylation of von Ebner's gland protein, and glycosylation of extracellular lacritin and proline-rich protein 1, with two different platforms (LC-electrospray ionization (ESI) MS/MS, and offline LC-MALDI MS/MS) to characterize the tear proteome. You et al. first identified glycosylation of lipocalin and cystatin Sandpin showed phosphorylation of nucleobindin 2 with 2DGE and specific dyes [74]. Zhao et al. characterized human tear phosphoproteins with the gel-based approach, and found lipocalin-1 phosphorylation at Ser24, and provided further information on the phosphorylation of lipocalin-1 at Ser32, Thr34, and Tyr36 [75]. Zhou et al. identified 67 N-glycosylated peptides from 43 unique proteins, 19 of which were discovered in tears [59]. A representative MS/MS analysis of an N-linked glycopeptide LSLHRPALEDLLLGSEAN#LTCTLTGLR ([M + 4H]^4+^, *m*/*z* = 741.9**,** N# = N-glycosylation site) derived from SNC66 protein in tear fluid of climatic droplet keratopathy was shown (Fig. 4), with an excellent b-ion and y-ion series (y_1_, y_3_, y_4_, y_5_, y_6_, y_7_, y_8_, y_9_, y_10_, b_10_^2+^, b_11_^2+^, b_12_^2+^, b_13_^2+^, and b_18_^2+^) and excellent S/N ratio. Perumal et al. presented a comprehensive analysis of the lacrimal proline-rich protein 4 (PRR4) with gel-based and gel-free methods [61, 62, 76]. Besides multiple splice variants, the acetylated, methylated, and oxidized forms of the proteins were also identified to show the extensive polymorphism of PRR4 and complicated mechanisms of modifications of human tear proteins [61, 76].

### Antimicrobial compounds in tears

The tear film directly contacts with the external environment, which is the first important barrier between external pathogens and the physical ocular surface epithelia. It has been evidenced by many studies that tears have the antimicrobial function at the ocular surface [78, 79]. Due to blinking and reflex tearing, the tear film can function in the following aspects: (i) physically scour the invading pathogens, and (ii) the abundant antimicrobial molecules in tears, which can distress pathogens or modulate epithelial innate responses to increase protection functions [80]. The “classic” tear antimicrobial components include mainly the following: (i) lysozyme in human tears, which is secreted by the lacrimal glands and accounts for about 20–30% of total tear protein content [47], and can kill Gram-positive bacteria [81]. The bactericidal mechanisms of lysozyme mainly catalyze hydrolysis of 1,4-beta-linkages between N-acetyl-D-glucosamine and N-acetylmuramic acid in the peptidoglycan backbone of a bacterial cytomembrane and also cleave chitodextrins in fungal cell walls, which has been reported to have anti-human immunodeficiency virus (anti-HIV) function [82]. (ii) Lactoferrin, with a great ability to bind divalent cations (such as iron), could remove the necessary nutrient for bacteria growth and even produce some toxins. Also, the iron-independent antimicrobial activities of lactoferrin have found that lactoferricin (a highly basic sequence at the N-terminus) could facilitate disruption of the cell membrane of bacteria, viruses, and fungi [83]. It is generally believed that lactoferrin has the function of bacteriostasis rather than bactericidal in the biological liquid such as tears [84]. (iii) Tear lipocalin, as the specific pre-albumin of tear fluid [85], accounts for up to 25% of the reflex tear, and is produced from the acinar cells of the main lacrimal gland [49]. Lipocalin, which has various isoforms in tears, has binds siderophores produced by a range of bacteria and thus plays a part in bacteriostatic activities by means of interfering pathogens to take up iron [49]. The previous studies have found that some lipocalin isoforms have a protease inhibitory domain and might protect the ocular surface from the cysteine protease’s attacks of microbial origin [86, 87]. (iv) sIgA, secreted by plasma cells (B lymphocytes) that are mainly derived from the lacrimal glands, accessory lacrimal glands, and conjunctival lymphoid tissues, is the primary antibody in tear film [88]. By virtue of its specific antigen-binding sites, sIgA can not only neutralize pathogens to prevent their attachment to host cells, but also unite with the lectin-like adhesin molecules in pathogens and latter removal in tear film. Consequently, sIgA is vitally important to remove pathogens from tear fluid where they enter the ocular surface [89]. (v) Low levels of functional compositions have also been detected in tears according to previous reports, including the relative amounts of various components (such as abundant C3 and factor B, but less C1q) [90, 91], the enzyme secretory phospholipase A 2 (sPLA2) [92], secretory leukocyte protease inhibitor (SLPI) [93], and surfactant protein (SP) A and D [94, 95]. However, it is worth noting that several of these molecules have other effects, and antimicrobial activities might not be their major function in tears [80].

### Autoantibody profiles in tear fluid

Normal human tear fluid contains multiple autoantibodies to endogenous tear proteins [96, 97]. Some study found that the levels of immunoglobulin are altered in diseased-eye tears [98, 99]. Moreover, the related lymphatic tissue exists in the lacrimal system [100]. Thus, tear antibodies might be derived from the blood, or generated from the immune tissue in the lacrimal system itself. It suggests that the alterations of specific proteins or autoantibodies play important roles in tear-related pathogenesis [101]. One study revealed a more-complex antibody repertoire against ocular antigens in dry-eye patients relative to controls, with western blot and multivariate statistical analyses [102], which might provide a clue to insight into the autoimmune mechanisms in some dry-eye pathogeneses [102]. Another studies found that anti-Ro/SSA and anti-La/SSB antibodies in tear fluid samples of Sjögren’s syndrome patients are associated with the severity of keratoconjunctivitis sicca [103], and that α-fodrin antibodies in tear fluid of Sjögren’s syndrome patients might be the activation markers of Sjögren’s syndrome (SS) [104]. Multiple studies demonstrated the involvement of the immune system in glaucoma [105, 106] and the alterations of autoantibody profiles in serum and aqueous humor of glaucoma patients, which can discriminate glaucoma patients from healthy individuals with high sensitivity and specificity [107].

## Disease-specific biomarker panels in tear fluid and their diagnostic potential

Proteomics and metabolomics research of body fluids present the most challenge for biomarker discovery of multiple diseases, and the study to discover biomarkers is mainly conducted in diverse body fluids [20, 108]. The main objective of biomarker discovery is to identify characteristic metabolites that can distinguish the disease with high specificity, high accuracy, and high sensitivity, and sequentially improve the diagnostic level. The second objective is to identify the pharmacological targets associated with the disease physiopathology, and thus significantly improve prognosis [20]. If the ideal biomarker is found in an easily accessible biological fluid, then the specific biomarker could be utilized in the healthy population [101]. Overall, it is critical to translate research advances of some diseases to clinical practice so that people could be real beneficiancer from the new developments, whether through new disease biomarkers or the development of advanced interventions [109]. However, there is still a large gap between the effort for biomarker discovery and the effective markers that are applied to clinical practice [110]. For the interested biomarker approach found in research to be adequately translated into the clinic application, multiple procedures must be followed, and include the discovery and determination of preclinical biomarkers, the validation of detection methods, the clinical qualification of that biomarker, assay development, and eventually the implementation via automated platforms for predictive, preventive, and personalized medicine (PPPM/3PM) [109]. Currently proposed biomarker panels in the tear fluid for ocular and systemic diseases are summarized (Table 2).

**Table 2:** Currently proposed biomarker panels in the tear fluid for ocular and systemic diseases

Disease	Specific molecular patterns	References
*Ocular diseases*		
DED	Lactoferrin	[68, 111–114]
Ocular allergies	IgE	[68, 114–120]
DED, SS, OGVHD	MMP-9	[121, 122]
DED, SS	IFN-γ	[123–127]
DED	TNF-α	[123, 128]
DED	IL-1α, IL-1β	[129, 130]
DED, SS	Th-17-associated cytokines (IL-6, IL-17A,IL-17F, and IL-22)	[123, 128, 131– 133]
DED	IL-8/ CXCL8	[123, 125, 126, 129]
SS, DED	MIP-1α/ CCL3, MIP-1β/CCL4	[129, 134]
SS, DED	RANTES/CCL5, Fractalkine/CX3CL1	[125, 135]
SS, DED	CXCL9, CXCL10, CXCL11	[130, 131, 136]
SS, DED	MCP-1/CCL2	[137]
DED	Lysozyme	[46, 48, 126, 129, 138]
DED	LCN-1 (lipocalin-1)	[21, 58, 63]
DED, SS, MGD	LPRR4, LPRR3, nasopharyngeal carcinoma-associated PRP4 and α-1 antitrypsin	[21, 29, 58, 63, 139]
DED	PIP (prolactin-inducible protein)	[59]
DED	S100 family of proteins: S100A8/calgranulin A, S100A9/calgranulin B, S100A4, and S100A11	[21, 58, 59, 62, 63, 139–141]
DED	Annexin A1 (ANXA1), annexin A11 (ANXA11)	[62, 63, 141]
DED, SS	MUC5AC	[133, 142–144]
SS	Cathepsin S	[133, 142–144]
DED	Neuromediators: substance P, NGF, VIP, CGRP	[12, 145]
***Systemic ocular and non-ocular diseases***		
DR	NGF, LCN-1, lactotransferrin apo A-I LCN-1, HSP27, B2M Endothelin, neuron-specific enolase	[146]
		[147]
		[146]
		[148]
MuSc	IgG Interleukin-2 α1-Antichymotrypsin	[32, 149, 150]
		[150]
		[151]
PD	TNF-α α-Synuclein	[152, 153]
		[153]
Choroidal melanoma	Cystatin C, lactoferrin	[154]
Breast cancer	Lacryglobin cystatin SA, 5-AMP-activated protein kinase subunit gamma-3, Triosephosphate isomerase, Microtubule-associated tumor suppressor 1 transferrin receptor protein 1, Putative lipocalin 1-like protein 1, DNA damage-binding protein 1; protein S100-A9, GTP-binding protein Di-Ras2 ALDH3A, TPI, C1Q1, S100A8 miR-21, miR-200c, exosomes	[155]
		[156]
Prostate cancer	Lacryglobin	[155]

### Advantages of tear fluid used to diagnose and predict chronic pathologic conditions

Whenever possible, noninvasive samples should be the highest priority. On this account, sputum, saliva, tears, sweat, and etc. are ideal sources because they could be obtained via noninvasive procedures [108]. In contrast, plasma, serum, or erythrocytes need specialized operator skills. Even the cerebrospinal fluid or a biopsy needs hospitalization, with high cost and poor practical applications for preventive population screening [137]. Furthermore, tears are proximal to the disease location (such as ocular surface disease and lacrimal gland disease) as compared with cancer biomarkers in blood, where the related biomarker molecules could be distant from the source, and are highly diluted [20]. In addition, urine has a good deal of interfering compounds (including cells, debris, and a high concentration of electrolytes) that must be removed before proteome analysis; and its preparation could result in protein loss and key disease-related information. Despite the fact that tear components are mainly derived from secretory glands such as the lacrimal glands, the change of tear film composition is not only regulated by its secretion units. Molecules can enter into the tear fluid through conjunctival vessels to reflect part-related information of systemic disease processes or other body sites. For example, because tear products could be affected by androgen levels as reported before, the tear film proteome might vary according to androgen-dependent diseases such as prostate cancer [157]. In the last few decades, the use of tear fluid to diagnose diseases has begun, and great achievements have been made without restriction to ocular diseases [109, 158, 159]. Therefore, proteomics studies for biomarker discovery in tear fluid are very promising areas for the treatment of local ocular diseases and systemic diseases.

### Ocular diseases

Dry-eye disease (DED), also called keratoconjunctivitis sicca (KCS), is a multifactorial disease of tear fluid and ocular surface, which would lead to various symptoms such as tear film instability, visual disturbance, and varying degrees of eye discomfort, and could be accompanied by potential damage to the ocular surface [160]. There are two main types of DEDs: evaporative dry eye (EDE) and aqueous tear-deficient dry eye (ADDE); the latter is divided into two major subclasses, Sjögren’s syndrome (SS) dry eye and non-SS dry eye [4, 160]. As one of the three fastest-growing eye diseases in the aging population, DED is quite underdiagnosed; only 10% of symptomatic patients have a clinically confirmed diagnosis. There is no “gold standard” for diagnosis and prognosis of DED that is partially due to the fathomless mechanisms of large number of this disease as well as the complexity and variability of tear fluid [109]. In addition, the mismatch between symptoms and clinical signs of DED makes diagnosis more difficult particularly in mild cases [140]. Therefore, the availability of biomarkers for the clinical application might represent a step forward in the progress of DED diagnosis and prognosis. The major biomarker classes of dry eye are as follows:

#### Translational biomarker patterns in tears

There are a few biomarkers approved by the US Food and Drug Administration (FDA) applied in the clinical setting to diagnose and treat DED [161]. In spite of numerous biomarker studies, only a tiny minority have entered into the clinical stages in ophthalmology. Three examples of proteins—lactoferrin, immunoglobulin E (IgE), matrix metalloproteinase 9 (MMP-9)—are successfully used as translational biomarkers, with diagnostic kits for multiple eye diseases that use the tear fluid as sample sources. (i) Lactoferrin, a primary iron-binding and multifunctional protein with immunomodulatory and antimicrobial functions, is consistently related to the aqueous-deficient DED [111]. Several publications have shown the significance of this marker; for example, a preliminary publication performed minimal ocular irritation in 156 DED patients, and the results demonstrated that the combination of lactoferrin and Schirmer's tests presents a great equilibrium between specificity and sensitivity [112]. Furthermore, when combined with the Schirmer's (the classical tear tests), tear lactoferrin presents high specificity (95%) and good sensitivity (72%) in the diagnosis of Sjögren's syndrome [113]. (ii) IgE, a protein with a crucial role in allergic diseases, is directly related to the activation of an allergic reaction on the ocular surface [115] and is associated with the critical immunological mechanism of allergic conjunctivitis [116, 117]. The confirmatory research of IgE that enrolled 124 patients with allergic conjunctivitis symptoms and 40 healthy patients as controls (*n* = 164) demonstrated that IgE level measurement could be an efficient approach to screen allergic conjunctivitis [118]. Some researchers also found that the detection of total IgE concentration is helpful for the clinical diagnosis of allergic conjunctivitis and even its severity [119, 120]. Furthermore, IgE and lactoferrin are applied to an advanced tear diagnostics method, called TearScan MicroAssay diagnostic tests [114]. If these biomarkers (IgE, and lactoferrin) are correlated with the dry eye, then TearScan MicroAssay diagnostic tests might assist in examination of the cause of DED, and help ophthalmologists to make a more-accurate decision [68]. (iii) Matrix metalloproteinase 9 (MMP-9) is a nonspecific inflammatory marker for inflammation-related ocular surface diseases, which is used as a tear protein biomarker in a diagnostic tool—the InflammaDry—for a clinician (RPSInc, Sarasota, FL, USA); namely, when MMP-9 is present in a small volume of tear sample [121], it will be captured by its specific monoclonal/polyclonal antibodies with an enzyme-linked immuno-sorbent assay (ELISA)-based sandwich immunoassay to display as a colored band [122].

#### Inflammatory cytokines

Contributing to the etiology of SS, DED, OGVHD, ocular allergy, and other inflammatory ocular surface diseases, immune-mediated mechanisms and inflammation are important regulation sections in these diseases. Some studies have demonstrated that various immune-related cytokines or chemokines in tears are notably increased in DED, OGVHD, ocular allergy, and other inflammatory conditions [123, 131, 134]. Besides the cytokines and chemokines, additional biomarkers have been applied in ocular allergy to estimate the extent of eosinophil, neutrophil, and lymphocyte infiltration, by means of investigating the contents of IgE, histamine, tryptase, and eosinophilic cationic protein (ECP) [140, 162]. In addition, the tear levels of these inflammatory molecules are interlinked with the disease severity and/or clinical indicators to further increase the feasibility of these molecules as potential biomarkers to evaluate diseases of ocular inflammation. For example, studies found increased levels of interferon-gamma (IFN-γ) in tear samples from DED and SS patients [124, 125]. Other studies also found elevated levels of tumor necrosis factor-alpha (TNF-α), which reflects the general inflammatory state of ocular surface disease in sub-sets of DED [128, 129]. TNF-α was also reported to be significantly increased in the tears of thyroid-associated ophthalmopathy (TAO) patients compared to controls [130]. Clinical studies reported that tears of dry-eye patients had increased levels of interleukin-1 alpha (IL-1α) and interleukin-1 beta (IL-1β), which were associated with corneal fluorescein staining [131]. Interleukin-17 (IL-17) and interleukin-22 (IL-22) act as effector cytokines of the T-helper 17 (Th-17) cells [132,163], which play a crucial part to maintain the chronic and relapsing phase of multiple immune diseases that include DED and SS, and were increased in DED patients compared to normal subjects [164]. Another significant cytokine, interleukin-6 (IL-6), was found in multiple studies, and might represent an effective biomarker to evaluate the therapeutic efficacy of ocular disease [165]. For example, Yoon et al. reported the elevated level of IL-6 in tears from dry-eye patients, and discovered that IL-6 was related to the severity of DED with Schirmer test, tear film break-up time (TBUT), goblet cell density, and other measures [128].

#### Inflammatory chemokines

Interleukin-8 (IL-8) is a crucial cytokine that directs migration of neutrophils, T-lymphocytes, and basophils, which was over-expressed in DED patient tears [123, 125, 129, 166]. A study found that IL-8 in combination with other inflammatory mediators (MMP-9, IFN-γ, interleukin-2 (IL-2), and epidermal growth factor (EGF)) could be typical biomarkers to assess the disease severity of DED [125]. Several studies of dry-eye patients demonstrated that multiple elevated tear chemokines, such as macrophage inflammatory protein 1 (MIP-1) beta/chemokine (C–C motif) ligand 4 (MIP-1β/CCL4), MIP-1 alpha/CCL3 (MIP-1α/CCL3), regulated the activation of normal T cells expressed and secreted CCL5 (RANTES/CCL5), chemokine (C-X-C motif) ligand 9 (CXCL9), CXCL10, CXCL11 fractalkine/chemokine (C-X3-C motif) ligand 1 (CX3CL1), and monocyte chemoattractant protein 1/CCL2 (MCP-1/CCL2), played significant roles in functions of monocytes and T-lymphocytes and were highly correlated with clinical parameters and disease severity [125, 126, 135, 136, 140, 167].

#### Protein biomarker panels

Because of high-sensitivity mass spectrometry in combination with additional validation techniques, proteome variations in the traces of tear proteins consistently have been reported in multiple studies [61–63, 77, 168]. As mentioned above, lactoferrin and lysozyme are known as crucial proteins in tears with antibacterial function to protect the ocular surface, and lipocalin is the key lipid-binding protein in tears. Several proteins, such as lysozyme-C, lipocalin-1 (LCN-1), lysozyme proline-rich protein 3 (LPRR3), lysozyme proline-rich protein 4 (LPRR4), prolactin-inducible protein (PIP), and nasopharyngeal carcinoma-associated PRP4 and α-1antitrypsin, were decreased in tears of DED, SS, and MGD ocular patients [59, 61, 63, 168]. Six proteins, including α-enolase, α-1 acid glycoprotein1 (AGP), S100A8/Calgranulin A, S100A9/Calgranulin B, S100A4, and S100A11 (Calgizzarin), were upregulated in tears of DED patients [59, 168, 169]. In addition, to search for proteome differences, tear proteins were analyzed via MS protein identification as well as network analysis, which revealed that various proteins are altered in tears, including S100A6, ceruloplasmin, phospholipase A2-activating protein (PLAA), cystatin S (CST4), secretoglobin family member 2A member 1, and albumin [58, 62, 63, 141]. Besides these extracellular proteins, multiple intracellular proteins, including annexin A1 (ANXA1), annexin A11 (ANXA11), aldehyde hydrogenase 3A1, and glutathione-S-transferase P1, were also reported to be deregulated in tears of DED patients [141, 170]. Other significant proteins, including mucin 5 subtype AC (MUC5AC) and cathepsin S, were altered in tears from DED patients [142, 171]. The previous studies found that the level of MUC5AC was decreased in the tears of SS patients with DED and non-SS DED patients, and correlated with an increased rate of inflammation [133, 143, 144]. As a lysosomal cysteine endopeptidase, cathepsin S was closely related to the immune responses, and it was proposed as a candidate biomarker for SS with its significantly elevated level in tears [171, 172]. Neuromediators such as substance P, vasoactive intestinal peptide (VIP), nerve growth factor (NGF), and calcitonin gene-related peptide (CGRP) were also analyzed with MS in tears [173, 174]. For example, the increased level of NGF in DED was correlated directly with disease severity, whereas, the decreased level of CGRP was correlated inversely with disease severity [145, 173].

It is evident that discovery of effective biomarkers in tear fluid has become a crucial focus in ocular surface disease research, and benefits from the easy access and advancement in analytical methodologies of tears. Furthermore, the development of tear collection, processing, and storage approaches also enables comparison across different studies and validates additional biomarkers from other analysis.

### Tear fluid protein biomarker panels in ocular and non-ocular systemic diseases

#### Diabetic retinopathy

Diabetic retinopathy (DR) is regarded as the most-common diabetic eye disease because of the perennial accumulated damage to small blood vessels in the retina to produce blindness [60]. Patients with diabetes have various patterns of tear proteins compared to healthy people [146, 175, 176]. One study found that levels of proinflammatory cytokines (such as IP-10 and MCP-1) were elevated, whereas the ratios of angiogenic cytokines and anti-angiogenic were decreased; those data might indicate angiogenesis and chronic inflammatory reaction on the ocular surface of diabetic patients [177]. In addition, NGF in tears exhibited a higher level in patients with proliferative diabetic retinopathy than that in non-diabetic controls and non-proliferative diabetic retinopathy patients; that parameter is promising to evaluate the status of DR [146]. Another study showed that the elevated level of the apolipoprotein A-I (apo A-I) in patients was accompanied by advanced diabetic retinopathy [147]. Moreover, 20 differentially expressed proteins were identified in tear samples of non-proliferative diabetic retinopathy patients [146]; and of them, heat shock protein 27 (HSP 27), LCN-1, and beta-2 micro globulin (B2M) present a progressive reduction, which might be biomarkers for early diabetic retinopathy [146]. Also, neuron-specific enolase and endothelin in tear fluid were increased in non-proliferative diabetic retinopathy patients [148].

#### Multiple sclerosis

The majority of studies of the tear proteins in multiple sclerosis disease focused on exploration of oligoclonal bands (OCBs) [32], which begins with the hypothesis that indicates that tears can reflect the microenvironment condition of the central nervous system (CNS) [151, 178]. The detection of oligoclonal IgG in cerebrospinal fluid (CSF) was one of the most serviceable tests to assist the diagnosis of multiple sclerosis [16, 149, 179], and there are several researchers who tried to ascertain the effect of OCBs in the tears of multiple sclerosis patients [179]. Two studies demonstrated that oligoclonal IgG bands can be detected in tears as well as in CSF of multiple sclerosis patients [16, 179]. Furthermore, one study discovered that soluble interleukin-2 levels were significantly increased in tears of multiple sclerosis patients compared to normal controls [150]. All these data account for the significance of tear compositions in the CNS and multiple sclerosis. A proteomics analysis on tear proteins from multiple sclerosis patients identified 185 tear proteins [151]. Among these differentially expressed proteins, alpha-1antichymotrypsin was the only one that was significantly increased in multiple sclerosis patients with ratio range of 1.6 to 2.5 (*p* < 0.05). Moreover, this increased protein in tears was also verified in CSF and serum via western blot and ELISA confirmed a relationship between tear fluid and CSF [151].

#### Parkinson’s disease

Parkinson’s disease (PD) is the most-common serious movement disorder in the world, and it is predominantly due to the selective loss of neurons in the substantia nigra to affect ~ 1% of adults older than 60 years [180]. Compared to other systemic diseases, studies on tear fluid biomarkers for PD are at a very early stage with only assessment of the quality and stability of the tear film in PD patients [181]. However, in 2013, a multiplex array analysis of tear fluids obtained from 18 PD patients compared to 17 healthy controls, which aimed to determine the TNF-α levels in tears and to explore the relationship between TNF-α and PD characteristics, discovered that the levels of TNF-α in PD patients were significantly higher than normal controls (*p* = 0.02) even though the level of TNF-α did not correlate with PD duration or severity [152]. This study also suggested that tear fluid was a suitable source to identify biomarkers and that TNF-α might be used as a marker of neurological inflammation signal in PD patients [152]. Furthermore, tears were proposed as a source of PD biomarkers [153]; the tears of 36 PD patients and 18 healthy controls were analyzed with a bottom-up LC–ESI–MS/MS workflow. Twenty-one significantly increased proteins and 19 significantly decreased proteins were found in PD patients compared to control groups. This prospective study might provide a supplement of proteomic alterations in PD and identify novel prospective tear biomarkers for PD diagnosis [153]. Another study analyzed the altered level of alpha-synuclein in tear fluid obtained from 75 PD patients, 75 healthy controls, and 31 atypical Parkinsonian patients with an ultra-sensitive single-molecule array (SIMOA) system and human alpha-synuclein immunoassay. Levels of total soluble alpha-synuclein in PD were significantly increased versus control subjects (*p* = 0.03; area under curve (AUC) PD versus controls 0.60) [182]. Alpha-synuclein could be detected and quantified in tears (small but significant differences) between PD and control subjects. Alpha-synuclein can be used as a promising source for further study of PD biomarker [182].

#### Cancers

Commonly, ocular tumors are not the primary tumor but the secondary metastases and growths from elsewhere in the body, particularly those from the breast, prostate, and bowel [183]. The only two kinds of ocular tumors which are primary tumors include retinoblastoma (usually affects children) and uveal melanoma (mainly affecting adults) [110]. Some studies found that tear protein biomarkers might exist for cancer in humans and dogs [184]. For example, ELISA analysis of cystatin C and lactoferrin in serum, tear fluid, and intraocular fluid samples of choroidal melanoma and benign eye tumors found that (i) for healthy controls, cystatin C concentration was significantly higher in serum than in tear and intraocular fluids; (ii) for choroidal melanoma patients, cystatin C concentration was similarly increased in tear fluids of both eyes; (iii) for healthy controls, lactoferrin was significantly higher in tears than in serum; and (iv) for benign and malignant eye tumors, lactoferrin was significantly elevated in tears [154]. These results clearly demonstrated that cystatin C and lactoferrin might be effective biomarkers to diagnose malignant and benign eye tumors.

Previous studies on breast cancer survival and mortality rate provide related evidence that earlier detection contributes to the drastically decreased mortality of breast cancer [185]. Unfortunately, small lesions are easily missed in fact and are not recognized even by mammography. Effective biomarkers in tears could be significant tools to improve the screening rate of breast cancer, even in patients with a precancerous lesion or at an early stage [137, 155]. For example, lacryglobin increases in human tear fluids from breast cancer and metastasis patients. An interesting study that evaluated tear samples from patients with different cancers with 1DE and 2DE approaches showed that lacryglobin in tears was present with different percentage of patients with colon (100%) and prostate cancer (100%), followed by cancers of the breast (88%), lung (83%), and ovary (33%), relative to controls (60%). Two control patients (60%) with lacryglobin presence had a family history of breast and prostate cancers [155]. Moreover, Lebrecht and his colleagues highlighted the significance of a biomarker in tear fluid to assist breast cancer patients successfully differentiated from healthy women with a specificity and sensitivity of ~ 90% with MS approach in serum and tears from 15 patients, which suggests that proteomics facilitates the discovery of new and effective biomarkers by analysis of tear fluid to diagnose breast cancer with high accuracy, sensitivity, and specificity [156]. Also, potential biomarkers screened in tears from 50 women with breast cancer and 50 age-matched healthy women. This diagnostic biomarker pattern differentiated cancer patients from healthy women with a specificity and sensitivity of ~ 70% [31]. A few years later, the Böhm group expanded differential tear protein analysis in breast carcinoma patients with MALDI-TOF-TOF semi-quantitative comparison proteomics, which found that some proteins were associated with various metabolic cascades such as triosephosphate isomerase (TPI) and aldehyde dehydrogenase 3A (ALDH3A), and host immune system pathways such as protein S100A8 (S100A8) and complement C1q subcomponent subunit C (C1Q1) [31]. A recent study found that breast cancer-specific miR-200c and miR-21 were highly expressed in tear exosomes in metastatic breast cancer patients when tear exosomes were analyzed between metastatic breast cancer patients (*n* = 5) and healthy controls (*n* = 8) with quantitative reverse-transcription polymerase chain reaction (qRT-PCR) and western blot [186], which clearly demonstrated that these two miRNAs were oncogenic miRNAs in tear exosomes in metastatic breast carcinoma patients, and that tear exosomes might be a biomarker source for diagnosis and prognostic assessment of metastatic breast cancers, and even other cancers [186].

Prostate cancer (PCa) is the second most-common cancer in men, and various factors such as family history, advanced age, low testosterone levels, diet rich in fats and BRCA1/2 mutations are associated with the cancer progression [187]. Although an increased level of prostate-specific antigen (PSA) is an effective indicator for PCa, its specificity and sensitivity as a screening marker for PCa still have considerable limitation and controversy. For example, the PSA test cannot distinguish among benign prostatic hyperplasia (BPH), non-aggressive, or aggressive PCa. Furthermore, PSA could give a false-positive or negative result on a PCa diagnosis [188, 189]. Therefore, it is important to identify more-effective biomarkers to achieve a more-accurate diagnosis and prognosis for PCa. MS analysis of the pooled tears from PCa patients and healthy controls found two characteristic peptides that missed with *m*/*z* 7110 and 14,213 Da in the tears of PCa compared to normal controls [190], and these two missed peptides should be sequenced in the PCa tear samples for development of novel PCa-specific biomarker panels**.**

## Most effective technologies for tear fluid biomarker panels’ analysis

Proteomic studies in tears have been carried out with various advanced techniques [64, 137, 153]. However, the analysis of tear fluid is still limited due to the high dynamic character of the tear proteome, as well as the small sample size [110]. Therefore, the protein content that can be used for analysis is low compared to blood or urine. Accordingly, very sensitive and specific detection methods are needed.

### Electrophoretic techniques and high-performance liquid chromatography

Proteins in tear fluids can be separated with gel electrophoretic methods that include 1DGE and 2DGE [102, 191–193], and the separated tear proteins can be applied for clinical diagnosis [194, 195]. 2DGE provides better protein separation compared to 1DGE. In the first dimension, proteins migrate to a specific area according to their isoelectric point (p*I*). In the second dimension, proteins are separated based on their relative molecular mass (*M*_*r*_). This two-step process enables identification of PTMs compared to 1DGE. For example, researchers exhibited a map of human tear protein PTMs with 2DGE in combination with a sequential staining workflow [74]. Quantification of a single protein in tears established a tear film protein map [176, 192, 196]. After the digital image analysis data files are created, multivariate statistical procedures are followed to compare the protein patterns of different test groups. Analysis of tear protein patterns with SDS-PAGE has also been explored to detect DED [168, 194, 197], DR [102, 146, 176], and blepharitis [29]. Despite its high sensitivity, 2D-SDS PAGE consumes high sample volume but displays low reproducibility, without any direct protein identification. The ability to discriminate was improved when fluorescent staining dye methods are used. The combination with differential in-gel electrophoresis (DIGE) could be a next step towards an effective diagnosis based on the tear patterns of patients [198].

Another crucial separation technique is high-performance liquid chromatography (HPLC), which is used to separate a mixture of compounds with a stationary phase. Depending on its outstanding ability to separate proteins in very small volume of tear samples, size-exclusion HPLC [197, 199], reversed-phase HPLC [138], ion-exchange HPLC [138], and nano-LC [51] have been so far applied to separate tear proteins.

### Microarray

The protein microarray platform is a highly accurate strategy based on proteomics to explore molecular variations at the protein level. In the past several years, protein microarrays such as antibody microarray have been extensively used to identify biomarkers in tear fluids [123, 127]. Briefly, to analyze the abundant changes of proteins in tears, specific antibodies are spotted onto nitrocellulose-coated slides, the tear proteins from patients and controls are labeled with fluorescence dye (Cy3, Cy5), and then the slides are incubated with the fluorescence dye-labeled tears to capture the corresponding antigens—tear proteins for comparison. This type of antibody microarray has been used to analyze the proinflammatory cytokine expression in the tears of DED patients and discovered a prominent difference between the different dry-eye subtypes [123]. The main advantage of this strategy is to simultaneously screen a large number of protein targets with high sensitivity and high specificity for the rapid confirmation of potential biomarkers for high-throughput clinical studies. In addition, it is a powerful tool to screen for high-quality MS-based protein biomarkers with easy operation. However, microarray technology is limited to the analysis of pre-selected target proteins, and is easily affected by cross-reactions and limited to the availability of commercial antigen/antibodies. Furthermore, microarray strategies are limited analysis of hydrophobic protein species such as membrane proteins. Due to the significant lipid-protein interactions in tear proteomics [21], proteomic strategies might be able to analyze more hydrophobic protein species.

### Mass spectrometry

In recent years, proteomics is developing rapidly with the aid of advanced MS techniques, and tear protein characteristics have been successfully analyzed with MS. Peptide mass fingerprints (PMF) based on MALDI-MS, and MS/MS based on ESI–MS, enable the high-throughput identification of proteins [200, 201]. Furthermore, MALDI-MS and ESI–MS can be combined with TOF or ion-trap analyzers. Those hybrid instruments, like ion-trap-Orbitrap analyzers (LTQ Orbitrap), have improved resolution and sensitivity [202, 203]. Furthermore, the nano-flow LC–MS/MS (nano-LC–MS/MS) can identify peptides with collision-induced fragment atom [204]. Nano-LC–ESI–MS/MS has been used to analyze proteins from tear samples of pterygium patients, and discovered higher levels of human alpha-defensins, S100A8/calgranulin A, and S100A9/calgranulin B, which could be used as indicators to predict recurrent pterygium [54, 169].

Moreover, prefractionation of the intricate proteome in tear scan has been realized with numerous separation strategies, which enables to capture a specific fraction of proteins in case of ion suppression effects because of the high complexity of protein samples [205]. However, the analysis of tear proteome is a quite challenging work. For example, study indicates that various types of protein modifications as well as point mutations occurred in tear proteins to make it difficult for tear protein analysis with conventional database-searching methods [53]. LC-MALDI-MS/MS is particularly suitable for PTM analysis, such as glycosylation and phosphorylation, and can be used to detect and characterize peptides with PTMs [53]. Another special MS technique for protein profiling analysis is SELDI-TOF–MS, which could detect more proteins (within the mass range of 1500 Da-30 kDa) than conventional electrophoretic separations [168, 195, 197]. This effective approach has been used in tear studies that uncover significant differences in tear protein profiles of DED patients [168] or breast cancer [156]. The high sensitivity and high specificity of this method not only are suitable for protein analysis from a small sample volume, such as tear fluid, but also are promising strategies to simultaneously analyze numerous patients. Accordingly, SELDI has become an attractive tool to analyze tear proteome in recent years [139, 168]; however, it is limited by the disadvantage that it has to handle the huge amount of protein data—thus, false positives are common, and this method only provides the molecular mass of the peptides which remains challenging to identify what specific protein the detected peak refers to. Furthermore, the large number of unassigned MS/MS spectra [206] and the lack of ionization for several peptide species [207] remain unsolved.

### ELISA

ELISA is an effective method to analyze tear components [208–210], which is the most-common approach for clinical examination to quantify tear proteins [211–213], and is a complementary method in some clinical studies [213, 214]. The setup of ELISA can be changed to quantitatively test different antigens and antibodies [208, 215, 216]. ELISA has been used to test the concentrations of IgA, IgG, and IgM in tears [213], quantify the dysregulated corneal dendritic cell density, nociception-associated factors, and vitamin D in tear fluids of evaporative dry eyes [208], and find that calcitonin gene-related peptides were elevated in tear fluid of migraine patients relative to normal controls [216]. Recently, a novel microfluidic paper-based analytical device (μPAD) and ELISA were used to analyze lactoferrin concentration in tears with symptoms of severe DED [209], which found that the abundance of tear lactoferrin in DED patients was positively correlated between μPAD and ELISA (*p* = 0.006, *r* = 0.886), and that tear lactoferrin concentration could reflect the severity of DED. Thereby, μPAD and ELISA are the effective methods to quantify tear lactoferrin in DED patients [209]. Another study found that tear dopamine was higher than in plasma dopamine, which suggested that tear fluid be a noninvasive source to monitor dopamine changes [217].

## Future perspectives

**Fig. 5:**
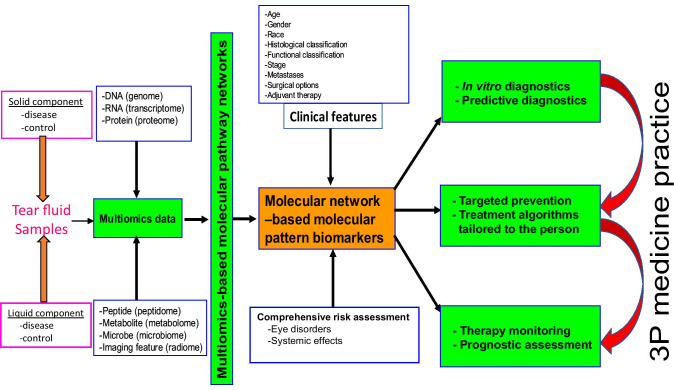
Scheme of multiomics, multiomics-based molecular pathway networks, and molecular network-based tear molecular pattern biomarkers for PPPM/3P medicine practice in different disease. Modified from Zhan X et al. [218], with copyright permission from Intech Open publisher, and from Li, Desiderio, and Zhan [219], with copyright permission from Wiley publisher

Human tear fluid includes solid components such as a few types of cells and liquid components such as complex biological compounds that include, but are not restricted to, lipids, proteins, peptides, metabolites, and electrolytes (Figs. 1 and 5) [20, 50, 51]. These tear component changes are extensively associated with ocular and/or systemic diseases [109]. Multiomics is an effective approach to identify and quantify those changes of tear fluid components in different pathophysiological conditions. As described in the “Composition of tear film,” “Disease-specific biomarkers in tear fluid and their diagnostic potential,” and “Most-effective technologies for tear fluid biomarker panels” sections, some progresses in human tear omics and biomarkers have been achieved [108, 161, 209]. However, it is necessary to strengthen the following studies in future.

### Strengthen the study on tear fluid multiomics-based molecular networks and biomarker panels in the framework of 3P medicine

Based on these solid and liquid components of tear fluid, different types of omics can be used to detect, identify, and quantify DNA (genomics data), RNA (transcriptomics data), protein (proteomics data), peptide (peptidomics data), metabolite (metabolomics data), microbe (microbiomics data), and image feature (radiomics data) (Fig. 5) [219, 220]. These omics data can be used to construct molecular pathway networks, and molecular network-based pattern biomarkers that are associated with different clinical factors (such as age, gender, race, histological classification, functional classification, stage, metastasis, surgical options, and adjuvant therapy) and high-risk factors (infection, inflammation, genetics, and life habit, etc.). These tear pattern biomarkers will be useful for prediction/prevention, diagnosis/therapy, and prognostic assessment in the framework of 3P medicine (Fig. 5) [219, 220].

### Emphasize the study on tear fluid phenomics in the framework of 3P medicine

**Fig. 6:**
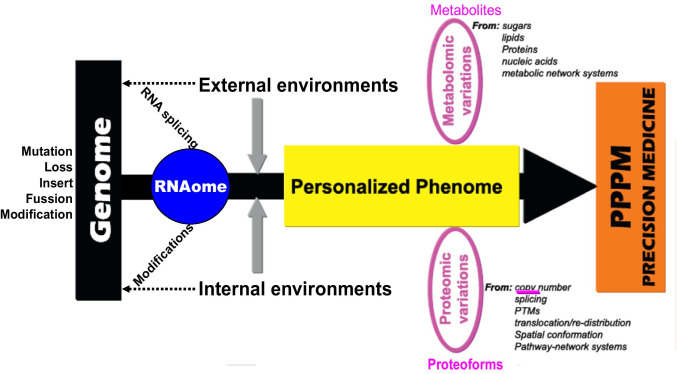
The unbalanced contributions of different tear omics to PPPM/3P medicine practice in different disease. PTMs = post-translational modifications. Modified from Zhan, Long, and Lu [221], with copyright permission from Elsevier publisher, and reproduced from Li, Desiderio, and Zhan [219], with copyright permission from Wiley publisher

Different omics have an imbalanced contribution to 3P medicine, namely individualized phoneme is the bridge from genome to 3P medicine (PPPM) or precision medicine (PM) practice, and metabolome and proteome are two main elements of phoneme (Fig. 6) [219, 221, 222]. The main contributions of genomics are the gene sequencing to detect, identify, and quantify mutation, loss, insert, fusion genes, and modifications in DNAs and RNAs. Proteomics and metabolomics are equally important in the research and clinical practice of PPPM/PM. Proteomic variations are involved in a series of changes, including copy number, alternative splicing, post-translational modifications, translocation/redistribution, and spatial conformation of proteins, and these proteins function in pathway-network system. Metabolomic variations are involved in the changes of all metabolites that are derived from sugar, lipid, protein, and nucleic acid, and these metabolites function in metabolic pathway-network system. Also, many metabolism processes are catalyzed by metabolic enzymes, and these metabolic enzymes still fit the basic features of proteins. Thus, current paradigm is shifting from genome-centered research practice to phenome-centered research practice (Fig. 6) [219, 221, 222]. Therefore, it is crucially necessary to strengthen study on tear phenomics, especially tear proteomics, metabolomics, and peptidomics, in ocular and/or systemic diseases, which will directly lead to the discovery of effective tear biomarkers to in-depth understand molecular mechanism of disease, and stratify patients for personalized prediction, diagnosis, and prognostic assessment of ocular and/or systemic diseases in the context of 3P medicine.

### Expand and strengthen the study on biomolecular modifications and proteoforms in tear fluid of ocular and/or systemic diseases

**Fig. 7:**
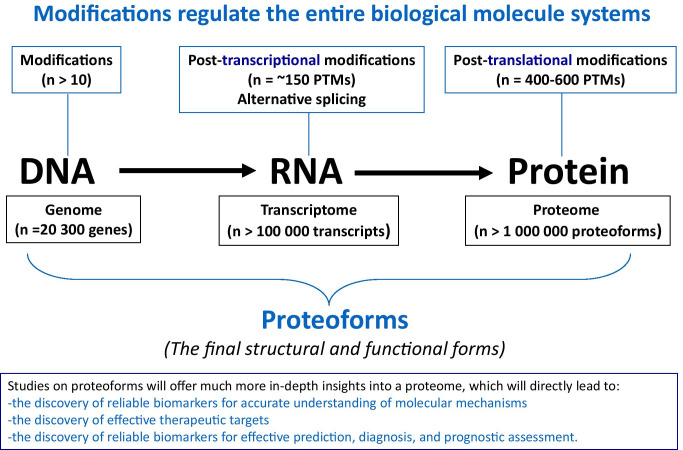
Modifications regulate the entire biological system at the levels of DNA, RNA, and protein, which finally results in the formation of proteoforms that are the final structural and functional forms of a gene or a protein. Constructed from Zhan, Long, and Lu [221], with copyright permission from Elsevier publisher, and constructed from Li, Desiderio, and Zhan [219], with copyright permission from Wiley publisher

**Fig. 8:**
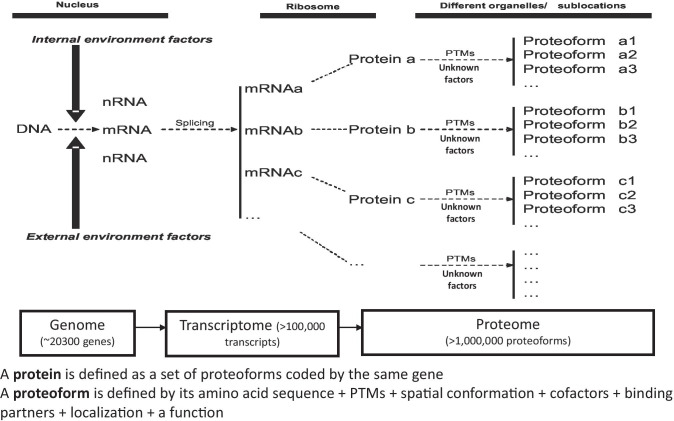
The concept and formation of proteoforms in tear fluid. PTMs = post-translational modifications. Modified from Zhan X et al. [223], with copyright permission from Hapres publisher, and from Zhan X et al. [220], with copyright permission from MDPI publisher

The information flow is transited from DNA, RNA to protein, and this process is systemically regulated by different modifications that occur in DNA, RNA, and protein (Fig. 7) [219, 221, 222]. About 10 types of modifications (such as DNA methylation) occurred in DNAs (~ 20,300 genes in human genome), ~ 150 types of post-transcriptional modifications (such as m6A methylation) and lots of alternative splicing occurred in RNAs (> 100,000 transcripts in human transcriptome), and 400–600 types of post-translational modifications (such as phosphorylation, glycosylation, ubiquitination, nitration, acetylation) occurred in proteins (> 1,000,000 proteoforms in human proteome). Proteoforms are the final structural and functional forms of gene and protein, which are the basic units of a proteome (Fig. 7) [219, 221, 222]. A proteoform is defined by its amino acid sequence + PTMs + spatial conformation + cofactors + binding partners + localization + a function, and a protein is defined as a set of proteoforms encoded by the same gene (Fig. 8) [220, 221]. This definition fully changed the traditional concept of a protein. Current research and practice regarding biomolecular modification and proteoforms are much insufficient in the field of multiomics, which is the future direction and will directly affect the entire medical and life sciences. Studies on proteoforms will offer much more in-depth insights into a proteome, which will directly lead to the discoveries of reliable biomarkers for accurate understanding of molecular mechanisms, effective therapeutic targets, and reliable biomarkers for effective prediction, diagnosis, and prognostic assessment (Figs. 7 and 8) [220, 221]. Therefore, there are significantly scientific merits in systematic study of biomolecular modifications and proteoforms in human tear fluids of ocular and/or systemic diseases.

### Emphasize the importance of mass spectrometry-based proteomics and metabolomics of tear fluid in understanding ocular and/or systemic diseases

As described above, proteome and metabolome are the essential elements of phenome (Fig. 6) [219, 221, 222]. Mass spectrometry (MS) is a crucial technology to identify protein amino acid sequence and abundance, modification site and abundance, and metabolite structure and abundance. Moreover, “top-down” MS and two-dimensional gel electrophoresis coupled with MS (2DE-MS) can provide super-high resolution power in identification of large-scale proteoforms. Recent years, four-dimensional MS (4D-MS; including retention time, *m*/*z* value, intensity, and ion mobility) [224] and data-independent acquisition (DIA) technologies such as sequential window acquisition of all theoretical spectra (SWATH) [225] are rapidly developing to significantly improve the capability to detect, identify, and quantify low-abundance and extremely low-abundance metabolites and proteoforms.

## Conclusions and expert recommendations in the context of 3P medicine

Human tear fluid demonstrates comprehensive profiles of biological compounds that include but are not restricted to electrolytes, metabolites, peptides, proteins, and lipids. Detailed qualitative and quantitative analysis of the molecular compounds in a tear fluid profile significantly improves identification and prediction of ocular disorders. On the other hand, the well-justified principle of a “sick eye in a sick body” makes comprehensive tear fluid biomarker profiling highly relevant also for advanced diagnostics and targeted treatment of systemic diseases. A prominent example is the Sicca syndrome linked to a cascade of severe complications that include DED and neurological and oncologic diseases [226–229]. The enormous socio-economic burden of severe non-communicable disorders to healthcare and society at large strongly motivated implementation of the global paradigm change from reactive to 3P medicine [230]. To this end, several health conditions benefiting from 3PM concepts and approaches have been exemplified in the literature for primary, secondary and tertiary care including sub-optimal health conditions, reversible damage to health, disease development, and progression [227, 229, 231–233].

Liquid biopsy, and in particular tear fluid analytical pathways, is considered to be optimal for the evidence-based predictive diagnosis, patient stratification, targeted treatment algorithms, treatment efficacy monitoring and prognosis, and cost-effective individualized prevention, which altogether are instrumental to implement the principles of 3P medicine in daily practice [234]. These principles are essential to follow also in case of pandemics such as the coronavirus disease 2019 (COVID-19) [235, 236].

In future, multiomics is an effective approach to elucidate precisely health status specific molecular patterns of DNA, RNA, peptides, proteins, and metabolites in the tear fluid of ocular and/or systemic diseases [237]. However, different omics demonstrate preferencial contributions to PPPM research and practice. The paradigm is shifted from genome-centered research practice to phenome-centered research practice in tear fluids, and of them, proteome and metabolome are two important aspects of the phenome [220, 221]. Further, complex modifications in DNA, RNA, protein, and metabolite profiles reflect the comprehensive regulation of the entire biological system [219]. Proteoforms are the final structural and functional forms of a gene and protein [220]. In-depth analysis of biomolecular modifications and proteoforms in tear fluids will directly lead to the discovery of effective biomarkers for patient stratification, personalized prediction/diagnosis, and prognostic assessment of ocular and/or systemic diseases in the framework of 3P medicine.

## Data Availability

Not applicable.

## Abbreviations

ADDE: Aqueous tear-deficient dry eye
AGP: α-1 Acid glycoprotein1
ALDH3A: Aldehyde dehydrogenase 3A
ANXA1: Annexin A1
ANXA11: Annexin A11
AUC: Area under curve
B2M: Beta-2 micro globulin
BPH: Benign prostatic hyperplasia
C1Q1: Complement C1q subcomponent subunit C
CID: Collision-induced dissociation
CGRP: Calcitonin gene-related peptide
CNS: Central nervous system
COVID-19: Coronavirus disease 2019
CSF: Cerebrospinal fluid
CST4: Cystatin S
DED: Dry-eye disease
DIGE: Differential in-gel electrophoresis
DMBT1: Malignant brain tumor 1 protein
DR: Diabetic retinopathy
ECP: Eosinophilic cationic protein
EDE: Evaporative dry eye
EGF: Epidermal growth factor
ELISA: Enzyme-linked immuno-sorbent assay
ES: Electrospray ionization
ETD: Electron transfer dissociation
FDA: Food and Drug Administration
HCD: Higher energy collision dissociation
HIV: Human immunodeficiency virus
HPLC: High-performance liquid chromatography
HSP27: Heat shock protein 27
IFN-γ: Interferon-gamma
IL-1α: Interleukin-1 alpha
IL-1β: Interleukin-1 beta
IL-2: Interleukin-2
IL-6: Interleukin-6
IL-8: Interleukin-8
IL-17: Interleukin-17
IL-22: Interleukin-22
IgE: Immunoglobulin E
IgG: Immunoglobulin G
IgM: Immunoglobulin M
KCS: Keratoconjunctivitis sicca
LC: Liquid chromatography
LCN-1: Lipocalin 1
LPRR3: Lysozyme proline-rich protein 3
LPRR4: Lysozyme proline-rich protein 4
MADI: Matrix-assisted laser/desorption ionization
MCP-1/CCL2: Monocyte chemoattractant protein 1/chemokine (C–C motif) ligand 2
MIP-1α/CCL3: Macrophage inflammatory protein 1 alpha/chemokine (C–C motif) ligand 3
MIP-1β/CCL4: Macrophage inflammatory protein 1 beta/chemokine (C–C motif) ligand 4
MGD: Meibomian gland disease
MUC5AC: Mucin 5 subtype AC
MMP-9: Matrix metalloproteinase 9
MS: Mass spectrometry
MS/MS: Tandem mass spectrometry
MuSc: Multiple sclerosis
NGF: Nerve growth factor
OCBs: Oligoclonal bands
OGVHD: Ocular graft-versus-host disease
PCa: Prostate cancer
PD: Parkinson’s disease
p*I*: Isoelectric point
PIP: Prolactin-inducible protein
PLAA: Phospholipase A2-activating protein
PMF: Peptide mass fingerprint
PPPM/3PM: Predictive, preventive, and personalized medicine
PRP4: Proline-rich protein 4
PSA: Prostate-specific antigen
PTMs: Post-translational modifications
qRT-PCR: Quantitative reverse-transcription polymerase chain reaction
RANTES/CCL5: Regulated the activation of normal T cells expressed and secreted chemokine (C–C motif) ligand 5
RP: Reverse phase
SCX: Strong cation exchange
SDS-PAGE: Sodium dodecyl sulfate–polyacrylamide gel electrophoresis
SELDI: Surface-enhanced laser desorption/ionization
sIgA: Secretory immunoglobin A
SIMOA: Single-molecule array
SLPI: Secretory leukocyte protease inhibitor
SP: Surfactant protein
sPLA2: Secretory phospholipase A2
SS: Sjögren’s syndrome
TAO: Thyroid-associated ophthalmopathy
TBUT: Tear film break-up time
Th-17: T-helper 17
TNF-α: Tumor necrosis factor-alpha
TOF: Time-of-flight
TPI: Triosephosphate isomerase
2D: Two dimensional
2DE: Two-dimensional gel electrophoresis
TOF: Time of flight
μPAD: Microfluidic paper-based analytical device
VIP: Vasoactive intestinal peptide
1DE: One-dimensional electrophoresis
